# Balloon-hooking technique for stabilizing a guiding catheter in tortuous supra-aortic vessel: A case report

**DOI:** 10.1016/j.radcr.2022.07.086

**Published:** 2022-08-16

**Authors:** Kenya Miyoshi, Yosuke Akamatsu, Daigo Kojima, Jun Yoshida, Yasushi Ogasawara, Hiroshi Kashimura, Yoshitaka Kubo, Kuniaki Ogasawara

**Affiliations:** a Department of Neurosurgery, Iwate Medical University School of Medicine, 2-1-1 Yahaba, Iwate, 028-3694, Japan; b Department of Neurosurgery, Iwate Prefectural Chubu Hospital, Kitakami, Iwate, Japan

**Keywords:** Balloon hooking, Giding catheter, Vertebral artery stenosis, Stenting

## Abstract

*Objective:* When performing endovascular interventions for supra-aortic vessels, the tortuous vascular anatomy observed in patients with atherosclerotic lesions often limits the ability to maintain a stable guiding catheter position. Here, we report a case of right vertebral artery (VA) stenosis treated with transfemoral stenting and discuss the utility of balloon-hooking technique using partially inflated balloon for stabilizing the balloon guiding catheter (BCG) in the supra-aortic vessel. *Case presentation:* A 74-year-old man who underwent right carotid artery stenting, coronary artery bypass grafting, and bilateral iliac artery stenting was admitted to our emergency department because of dizziness related to head movement. Computed tomography angiography revealed right VA origin stenosis and left subclavian artery (SA) occlusion. The patient underwent stenting of the right VA. After several unsuccessful cannulation attempts into the right VA through transradial access, transfemoral access was obtained through the left iliac stent. A 9-Fr BGC was navigated into the right SA. The balloon was partially inflated just distal to the first curve of the right SA and used as hook by pulling back until the proximal edge of the balloon was pushed distally by the lesser curvature of the SA, resulting in stabilization of the BGC and successful angioplasty and stent deployment at the VA stenosis. The patient's symptoms resolved completely, without any neurological deficits. *Conclusions:* Balloon-hooking technique using a partially inflated BGC is feasible for stabilizing the guiding catheter in tortuous supra-aortic vessel.

## Introduction

Steno-occlusive disease of subclavian artery (SA) or vertebral artery (VA) origin can be treated with either extra-thoracic bypass surgery or endovascular treatment, although endovascular therapy is performed more frequently [1], [2], [3], [4]. However, atherosclerotic lesions frequently linked to vascular tortuosity can become a limiting factor for endovascular therapy due to difficulty of stable placement of guiding catheters [5]. In cases of steno-occlusive lesions of the VA, transradial/brachial, transfemoral, combined transradial, and transfemoral approaches have been used [6,7]. However, proper placement of the guiding catheter through transfemoral access is often difficult because of the short landing zone of the proximal SA. Furthermore, the acute angle from the SA to the right VA impedes catheterization of the right VA, even through trans-radial/brachial access [8]. Transbrachio-femoral approach, or so-called pull-through technique, has also been applied to stabilize the guiding catheter [9,10]. However, because another arterial puncture, catheter placement and handling of snare devices are required and may increase the procedure time and risk of access site complications and endothelial damage caused by the wire being pulled from 2 different access sites, availability of alternative technique without the pull-through is beneficial. Balloon guiding catheters (BGCs) have also been used for anchoring guiding catheter with fully inflated balloon in the target vessel [11].

Here, we report a case treated with transfemoral stenting of right VA origin stenosis and discuss the utility of balloon-hooking technique using partially inflated balloon for stabilizing the BCG in the supra-aortic vessel.

## Case presentation

**Fig. 1:**
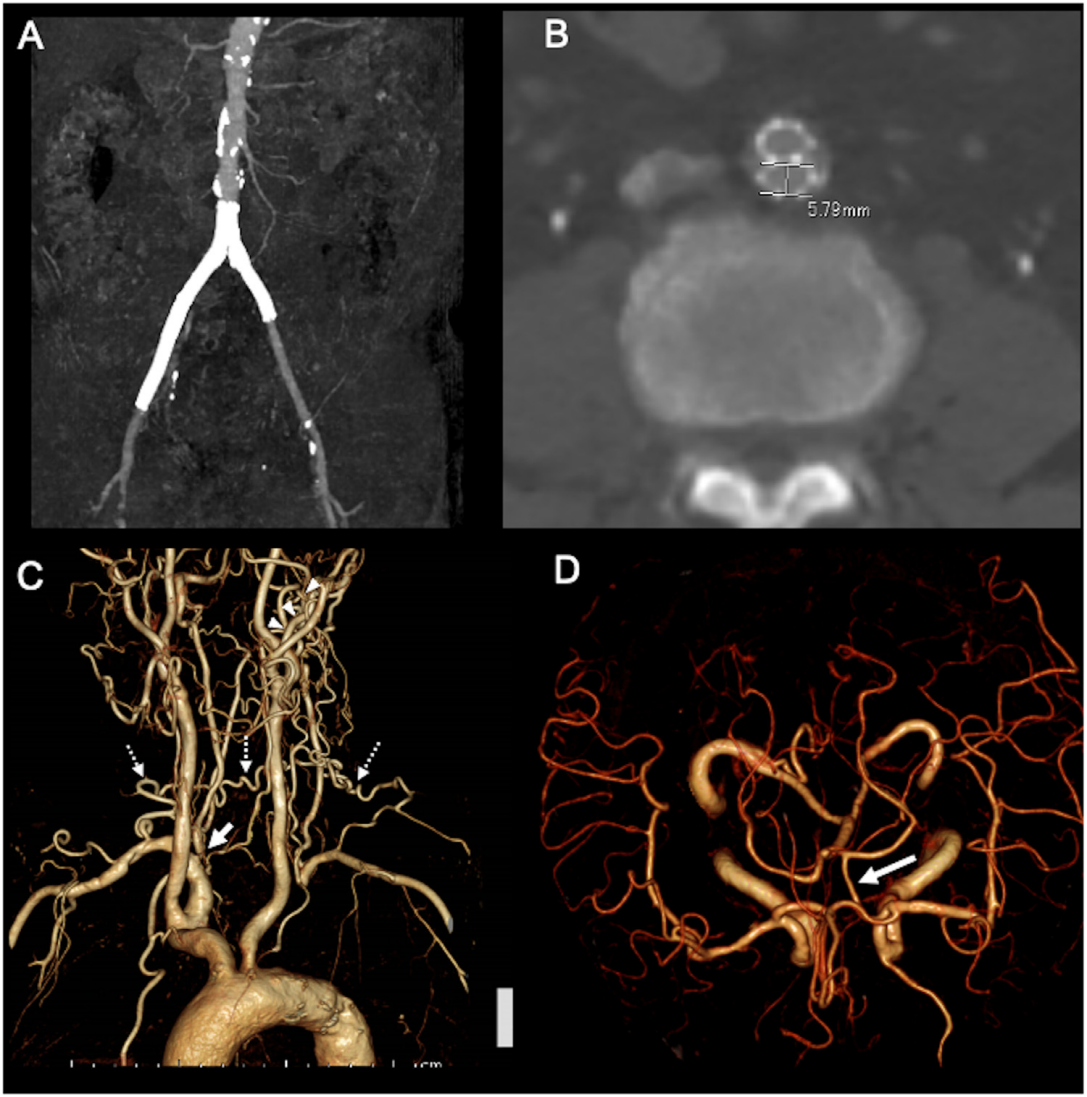
(A) Computed tomography angiography (CTA) demonstrates two stents in both the iliac arteries. (B) Cross-sectional image of the CTA shows the diameter of the stent (5.79 mm), which is larger than the outer diameter of the 9-Fr guiding catheter (3.0 mm). C) CTA of the supra-aortic vessels shows right vertebral artery origin stenosis (arrow), left subclavian artery occlusion, and robust collaterals of the bilateral deep cervical (dotted arrows) and occipital arteries (arrowheads). (D) Intracranial CTA shows communication between the left posterior communicating artery and ipsilateral P1 segment of the posterior cerebral artery.

A 74-year-old man with a history of right carotid artery stenting, coronary artery bypass grafting, and bilateral iliac artery stenting presented to our hospital with a 3-month history of feeling faint when tilting his head to the right side. Neurological examination was normal except for feeling faint when he tilted his head to the right; the symptoms resolved soon after his head returned to a neutral position. Because the symptoms occurred when sleeping in the right lateral position, the patient developed excessive fear, and he became sleep-deprived. CTA revealed bilateral iliac artery stents (Figs. 1A and B) and stenosis of the right VA and left SA occlusion, and multiple robust anastomoses from the bilateral deep cervical and occipital arteries were observed (Fig. 1C). Communication between the left posterior communicating artery and the ipsilateral P1 segment of the posterior cerebral artery was also visualized on CTA (Fig. 1D).

Based on the clinical and radiological findings, we concluded that the stenotic right VA, which was the dominant artery of the posterior circulation, was more constricted by head tilting. Thus, reduced blood flow to the posterior circulation caused the light-headed feeling in this patient. The decision was made to recanalize the right VA.

**Fig. 2:**
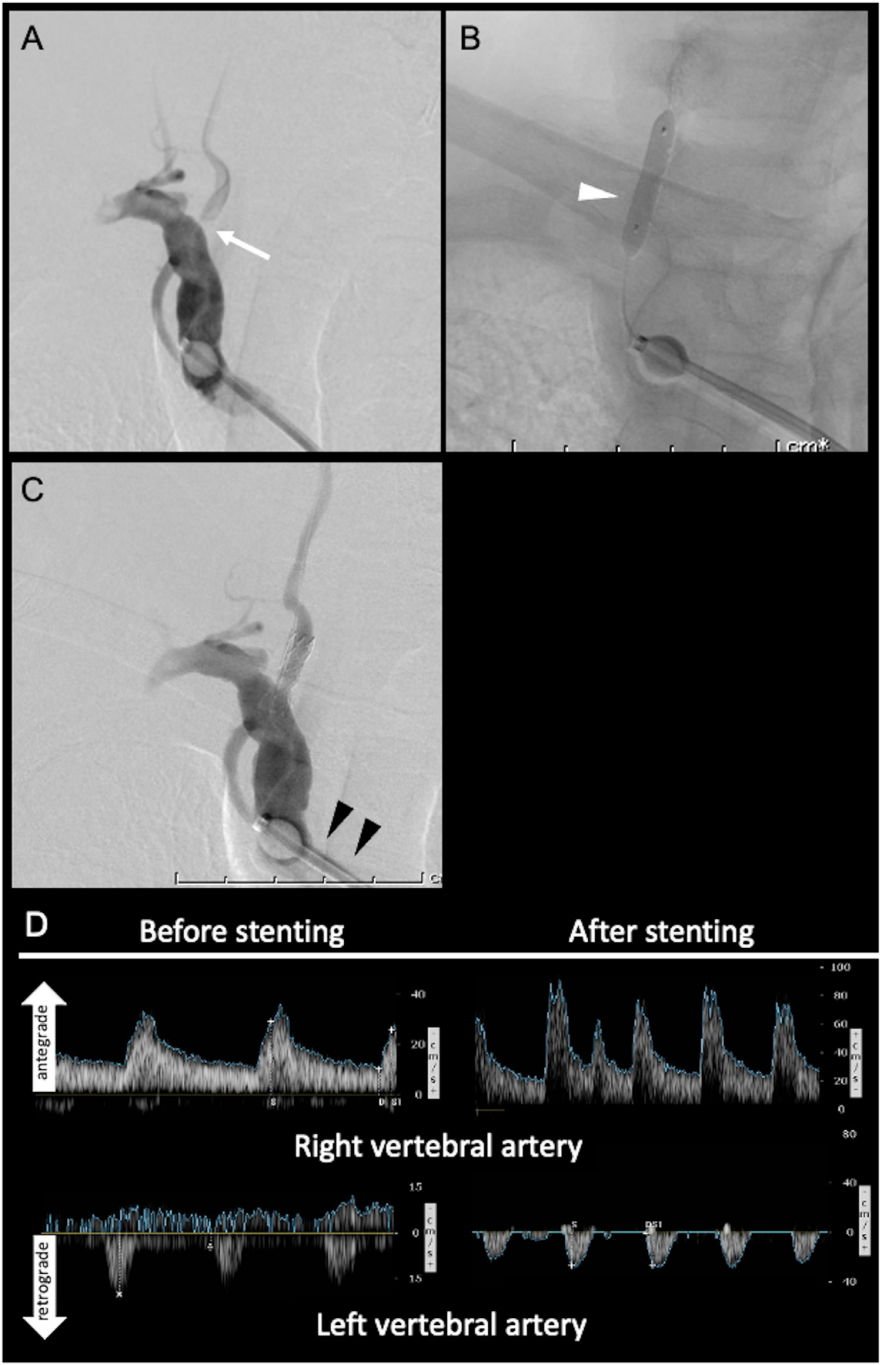
(A) Right subclavian artery injection demonstrates severe stenosis of the right vertebral artery (white arrow) and retrograde filling of the subclavian artery through the partially inflated balloon (arrowheads). B) Non-subtracted image shows non-compliant balloon inflated over the stenotic lesion (white arrowhead). (C) Right subclavian artery injection shows sufficient dilatation of the right vertebral artery. Note that retrograde filling was not observed because of the balloon in the subclavian artery (black arrowheads). (D) Doppler ultrasonography of the right and left vertebral artery obtained before and after the stenting showing increase in antegrade flow of the right vertebral artery.

He was preloaded with aspirin (100 mg/daily) and clopidogrel (75 mg/daily) for seven days prior to the procedure. Our first attempt to navigate the guidewire into the right VA through transradial access failed because of acute angulation of the VA origin stenosis from the SA. Therefore, a 9-Fr sheath was placed in the left femoral artery and navigated through the left iliac stent, and a 9-Fr BGC (Optimo, Tokai Medical Products, Aichi, Japan) was inserted into the right SA over a wire and a 6-Fr intermediate catheter. Because the short landing area of the guiding catheter in the SA restricted stabilization of the guiding catheter in the proper position, the balloon was partially inflated just distal to the first curve of the right SA and pulled back until the proximal edge of the balloon was pushed distally by the lesser curvature of the SA, resulting in stable positioning of the BGC (Fig. 2A). During the subsequent procedure, the balloon was also used as distal protection device by temporary balloon occlusion of the SA. A non-compliant balloon (Aviator Plus, 4 × 20 mm, Cordis Corporation, NJ) was navigated over a 0.014 wire (Synchro Select Support, 200 cm, Boston Scientific, Natick, MA) and inflated for angioplasty (Fig. 2B). A balloon-expandable stent (PALMAZ Genesis 5 × 18 mm, Cordis Corporation) was deployed over the lesion, resulting in satisfactory expansion of the lesion (Fig. 2C). Carotid ultrasonography showed improvement in the antegrade flow of the right VA (Fig. 2D). The patient's symptoms resolved completely, and he was discharged without any neurological deficits. The step-by-step procedure is shown in Supplementary Video 1.

## Discussion

We demonstrated the utility of balloon-hooking technique using the partially inflated BGC for stabilizing the BGC at the proximal SA in a case of transfemoral stenting of right VA origin stenosis.

Although stable placement of guiding catheters is mandatory for endovascular treatment, the short and tortuous landing area of the guiding catheter can lead to unstable positioning of the guiding catheter when performing transfemoral endovascular treatment for the proximal VA, subclavian artery, or brachiocephalic artery. In this setting, the so-called pull-through technique has been used for stabilizing a guiding catheter [12]. However, a guiding wire should be passed between 2 access sites with the support of a catheter or snare device in the standard pull-through technique, which is sometimes challenging and may increase the procedure time [9,13]. Furthermore, because navigation of another wire non-coaxially with a guide wire for stenting requires a large bore sheath with an outer diameter of at least 10 Fr, access site complications are a concern in patients on antiplatelet therapy [14]. In contrast, our balloon-hooking technique has several advantages. First, stable positioning of the guiding catheter can be achieved with a single placement of a partially inflated BGC in the SA. Second, lesion cross of the steno-occlusive VA can be performed under proximal protection by temporary occlusion of the SA. Moreover, the entire procedure can be completed under cerebral protection if ischemic tolerance against balloon occlusion of the SA is confirmed, as in the present case. However, if the patient does not tolerant temporary occlusion of the SA, placement of a filter device in the distal VA segment should be considered as a distal protection device.

When performing balloon-hooking technique, the BGC has to be navigated beyond the lesser curvature of the target vessel. The partially inflated BGC was then pulled back until the proximal edge of the balloon was pushed distally by the lesser curvature of the target vessel. The present technique is different from balloon-inflation anchoring technique enabling stabilization of the BGC in the target vessel with fully inflated balloon [11].

We successfully treated a stenotic lesion of right VA origin through a partially inflated BGC. The use of a partially inflated BGC like a hook was useful for stabilizing the guiding catheter at the proximal SA in patients with VA origin stenosis. Further cases are needed to establish its utility.

## Human rights statements and informed consent

All procedures followed were in accordance with the ethical standards of the responsible committee on human experimentation (institutional and national) and with the Helsinki Declaration of 1964 and later versions. Informed consent was obtained from the patient for publication of this case report and any accompanying images.

## Patient consent

Informed consent was obtained from the patient for the publication of this case report.

## Appendix Supplementary materials

Image, video 1
